# An induced population of *Trypanosoma cruzi* epimastigotes more resistant to complement lysis promotes a phenotype with greater differentiation, invasiveness, and release of extracellular vesicles

**DOI:** 10.3389/fcimb.2022.1046681

**Published:** 2022-12-14

**Authors:** Izadora Volpato Rossi, Maria Alice Ferreira Nunes, Bruna Sabatke, Hennrique Taborda Ribas, Sheila Maria Brochado Winnischofer, Augusto Savio Peixoto Ramos, Jameel Malhador Inal, Marcel Ivan Ramirez

**Affiliations:** ^1^ Graduate Program in Cell and Molecular Biology, Federal University of Paraná, Curitiba, PR, Brazil; ^2^ Carlos Chagas Institute, Fundação Oswaldo Cruz (FIOCRUZ-PR), Curitiba, PR, Brazil; ^3^ Graduate Program in Microbiology, Pathology and Parasitology, Federal University of Paraná, Curitiba, PR, Brazil; ^4^ Graduate Program in Biochemistry Sciences, Federal University of Paraná, Curitiba, PR, Brazil; ^5^ Department of Biochemistry and Molecular Biology, Federal University of Paraná, Curitiba, PR, Brazil; ^6^ School of Human Sciences, London Metropolitan University, London, United Kingdom; ^7^ School of Life and Medical Sciences, University of Hertfordshire, London, United Kingdom

**Keywords:** *Trypanosoma cruzi*, complement system, infection, extracellular vesicles, Chagas disease

## Abstract

**Introduction:**

Chagas disease is a neglected tropical disease caused by *Trypanosoma cruzi*, which uses blood-feeding triatomine bugs as a vector to finally infect mammalian hosts. Upon entering the host, the parasite needs to effectively evade the attack of the complement system and quickly invade cells to guarantee an infection. In order to accomplish this, *T. cruzi* expresses different molecules on its surface and releases extracellular vesicles (EVs).

**Methods:**

Here, we have selected a population of epimastigotes (a replicative form) from *T. cruzi* through two rounds of exposure to normal human serum (NHS), to reach 30% survival (2R population). This 2R population was characterized in several aspects and compared to Wild type population.

**Results:**

The 2R population had a favored metacyclogenesis compared with wild-type (WT) parasites. 2R metacyclic trypomastigotes had a two-fold increase in resistance to complementmediated lysis and were at least three times more infective to eukaryotic cells, probably due to a higher GP82 expression in the resistant population. Moreover, we have shown that EVs from resistant parasites can transfer the invasive phenotype to the WT population. In addition, we showed that the virulence phenotype of the selected population remains in the trypomastigote form derived from cell culture, which is more infective and also has a higher rate of release of trypomastigotes from infected cells.

**Conclusions:**

Altogether, these data indicate that it is possible to select parasites after exposure to a particular stress factor and that the phenotype of epimastigotes remained in the infective stage. Importantly, EVs seem to be an important virulence fator increasing mechanism in this context of survival and persistence in the host.

## 1 Introduction

*Trypanosoma cruzi*, the causative agent of Chagas disease, is a hemoflagellate protozoan that has a complex life cycle, transiting between hematophagous triatomine vectors and a mammalian vertebrate host. In the insect, replicative epimastigotes (EPIs) convert to infective metacyclic trypomastigotes (METAs) that move from the midgut to the hindgut of the vector. The infective metacyclic trypomastigotes are released in the excreta during the insect’s blood meal and can enter a mammalian host through wounds in the skin or mucous membranes (Melo et al., 2020). The METAs face two major challenges in the host: evading immune system attack and rapidly invading mammalian cells (I. Cestari and Ramirez, 2010). Once inside the cell, they replicate as amastigotes and are released as bloodstream trypomastigotes, which can be obtained experimentally *in vitro* as tissue culture-derived trypomastigotes. The infective forms of *T. cruzi* face the challenge of evading the immune system and promoting infection in host cells (De Souza et al, 2010).

One of the early mechanisms of innate immunity is the activation of the complement system, which can result in the direct killing of microorganisms and enhance their clearance by phagocytes (Lambris et al., 2008). It is known that METAs, bloodstream trypomastigotes, and also amastigotes present an increased resistance to complement-mediated lysis compared with EPIs (Nogueira et al., 1975; Kipnis, 1985; Iida et al., 1989). During the differentiation from epimastigote to metacyclic trypomastigote in the insect vector, the parasite undergoes a series of morphological and physiological changes that confer the capacity to evade complement-mediated killing (Lidani et al., 2017). The mechanisms controlling this resistance seem to be multifactorial, involving the expression of complement receptors on their surface, such as trypomastigote decay-accelerating factor (Tambourgi et al., 1993), complement regulatory protein (Norris, 1998), calreticulin (Ferreira et al., 2004), and CRIT (I. dos S. Cestari et al., 2008). As an intracellular parasite, the success of the infection with *T. cruzi* is to evade the complement system and invade host cells, a process that requires different strategies (Yoshida, 2006; Cestari and Ramirez, 2010).

In recent years, it has been described that the release of extracellular vesicles (EVs) is used as a cell–cell communication strategy during trypanosomatid infection (reviewed by Rossi et al., 2021). EVs are complex lipid bilayer structures that transport various molecules derived from the source cell, such as proteins and nucleic acids (Gavinho et al., 2018; Margolis and Sadovsky, 2019), and are currently classified into exosomes or microvesicles according to their biogenesis (Théry et al., 2018). Our group demonstrated that EVs derived from the interaction of *T. cruzi* with host cells are able to inhibit the lysis mediated by the complement system and increase the infectivity of the parasites (Cestari et al., 2012; Ramirez et al., 2017; Wyllie and Ramirez, 2017).

*Trypanosoma cruzi* has a well-recognized genetic and phenotypic heterogeneity, which results in a complex mosaic of subpopulations (Macedo et al., 2004; Lewis et al., 2009; Zingales et al., 2012; Seco-Hidalgo et al., 2015; Belew et al., 2017; Jimenez et al., 2019). This variety may confer an evolutionary advantage when dealing with a parasite that needs to face and survive the constant change of scenarios, such as exposure to the host’s immune system, nutritional supply, different tissues, etc. Here, we hypothesized whether exposure of epimastigote forms to normal human serum (NHS) would be able to select more resistant parasites, and we decided to follow the phenotype of this population between the different stages of the pathogen. To investigate, we performed a survivor selection protocol with two rounds of short exposure of the parasites to NHS, and we evaluated the selected population for its resistance to complement attack, the rate of metacyclogenesis, and its subsequent invasiveness. We showed that the selected population was more resistant to complement lysis and also more infectious to mammalian cells. We also have seen that the virulence phenotype of the selected population remains in the trypomastigote form derived from cell culture, which had a higher rate of parasite release from infected cells. EVs derived from the selected population were capable of increasing the invasion of WT parasites and could modulate the dynamics of infection and release of tissue culture-derived trypomastigotes (TCTs) from infected cells. This evidence supports the idea that the parasite during its life cycle activates a specific expression of molecules and transient mechanisms to respond and resist the immune innate system, invading cells and promoting infection.

## 2 Methods

### 2.1 Parasite and mammalian cell culture

Human monocyte THP-1 and African green monkey kidney Vero cell lines were grown in RPMI 1640 medium supplemented with 10% fetal bovine serum (FBS) and penicillin–streptomycin (100 IU/ml, 100 mg/ml) in a humid atmosphere of 5% CO_2_ at 37°C. *Trypanosoma cruzi* epimastigote forms (G strain) were cultured in liver infusion tryptose (LIT) medium supplemented with hemin at 10 mg/ml and 10% FBS at 28°C as previously described (Camargo, 1964). METAs were obtained by differentiation of 1 × 10^8^ epimastigotes from cultures at the stationary growth phase after incubation for 10 days in 15 ml of TC100 insect medium (Vitrocell, Campinas, Brazil) in a mixture with 5 ml of complete LIT medium in 75 cm^2^ flasks. As the induction of metacyclogenesis involves a stimulus by nutritional starvation (approximately 2.5% FBS), the viability of the parasites during differentiation was evaluated by counting parasites with normal morphology and movement on different days after the onset of metacyclogenesis. Trypan blue 0.4% staining was made in parallel to validate viability under optical microscopy. Metacyclic trypomastigotes were purified by passage through a diethylaminoethyl-cellulose column, as described by Teixeira and Yoshida (1986). TCTs were collected from the culture supernatants of infected Vero cells.

### 2.2 Parasite subpopulation selection

Log phase EPIs (6 × 10^5^/ml, G strain) were exposed to 30% NHS in LIT medium (v/v) at 37°C. The lysis of the population was assessed by counting in a hemocytometer chamber every 2 min. Parasite death was stopped when it reached only 30% of survivors (taking about 20 min) by adding cold LIT medium followed by centrifugation at 1,000×*g*. The remaining population was recovered upon completion of a regular growth curve and was named 1R. These 1R parasites were again exposed to lysis under the same conditions (30% NHS until 30% survival), and the remaining population was recovered and named 2R.

### 2.3 Epimastigote growth curve in NHS serum

The growth curve was initiated at a density of 5 × 10^5^ epimastigotes/ml. To assess the growth rate in the presence of NHS, 5% NHS (v/v) was added to the LIT medium. As control, parasites were grown in NHS-free LIT. Parasites were counted every 24 h in a hemocytometer chamber.

### 2.4 Invasion assays

Vero cells from logarithmically grown culture were treated with trypsin, washed once with RPMI 1640, and seeded on 13-mm coverslips in 24-well plates (1.0 × 10^5^ cells per well) until semi-confluence was reached (16 to 24 h). After this, cells were washed with serum-free RPMI and incubated with *T. cruzi* metacyclic (META) or TCT forms at a multiplicity of infection (MOI) of 10 for 180 min at 37°C in 5% CO_2_. Cells were then washed with RPMI, fixed with 4% paraformaldehyde for 15 min, washed with RPMI, and stained with Giemsa diluted 1:10 for 15 min at room temperature. Then, they were washed with H_2_O and slides were mounted with the mounting medium Entellan (Merck, Darmstadt, Germany). Intracellular parasites were quantified by light microscopy, counting at least 300 cells per slide. For invasion assays in the presence of NHS, cells were seeded as described above, and parasites were added in the presence of different concentrations of NHS (diluted in serum-free RPMI 1640), followed by 180 min of incubation at 37°C with 5% CO_2_. As control, parasites were added to wells containing Vero cells without NHS. Staining and quantification were performed as described above.

### 2.5 Transwell experiments: Simultaneous EV release and cell invasion

Transwell^®^ systems (Corning, New York, United States of America) containing inserts with a 0.45-μm membrane pore size were used. Vero cells (1 × 10^5^ cells/well) were seeded on 13-mm coverslips in the lower chamber (overnight at 37°C with 5% CO_2_ for adherence) of 24-well plates. Prior to infection, Vero cells were washed with serum-free RPMI. At the time of the assay, THP-1 cells in interaction with METAs from the WT or 2R population (10:1, 5 × 10^6^ parasites to 5 × 10^5^ THP-1 cells) were incubated in the upper chamber. At the same time, WT metacyclic trypomastigotes (1 × 10^6^) were added to the lower chamber for the invasion assay in a parasite/cell ratio of 10:1. As control, it was used as medium or THP-1 cells (5 × 10^5^) in the upper chamber. CaCl_2_ (1 mM) was added to each upper insert, and the plate was incubated for 3 h at 37°C. Vero cells were then washed with serum-free medium and fixed and stained with Giemsa as described above. Intracellular parasites were quantified by light microscopy, counting at least 300 cells per slide. The *x*-axis labels indicate what was placed in the upper chamber of the Transwell plate.

### 2.6 Complement-mediated killing assays

The NHS collected from non-Chagasic healthy voluntary donors was obtained and stored at −80°C until needed. The complement activity of serum samples was evaluated by determining the dilution of serum required to lyse approximately 50% of parasites (limiting dilution assay). Controls included parasites incubated in RPMI medium.

Log-phase EPIs were washed (500×*g*, 10 min) in RPMI medium and resuspended at a concentration of 5.0 × 10^6^/ml. Parasite suspension (100 μl, 5.0 × 10^5^) was incubated in 100 μl of NHS in different concentrations for a certain time at 37°C. Reactions were stopped by the addition of 800 μl of ice-cold RPMI. Surviving parasites were quantified in a hemocytometer chamber by optical microscopy (×40 objective) based on morphology integrity and movement, being determined by the percentage of parasite death.

For complement-mediated lysis in the presence of EVs, parasites (5.0 × 10^5^ in 100 μl) were incubated with 100 μl of 6.25% NHS with or without EVs (5 μg) for 10 min at 37°C. Reactions were analyzed as mentioned above.

### 2.7 EV production and characterization

The EVs were obtained using a protocol adapted from Cestari et al. (2012). The EVs were isolated from the interaction of *T. cruzi* parasites with THP-1 monocytic cells in a 5:1 ratio. To stimulate EV release, THP-1 cells (1 × 10^6^/ml) were incubated with parasites (5 × 10^6^/ml) for 1 h in a serum-free RPMI medium including 1 mM of CaCl_2_ at 37°C. After stimulation, the EVs were collected from the supernatant and isolated by differential centrifugation as follows: 500×*g* for 5 min, 4,000×*g* for 30 min to eliminate debris, and 100,000×*g* for 90 min to obtain an EV pellet containing EVs of both the parasites and THP-1. Ultracentrifugation was carried out using a Beckman Coulter Optima MAX-XP ultracentrifuge with an MLS-50 aluminum swing-out rotor. Pellets were resuspended in cold phosphate-buffered saline (PBS).

### 2.8 EV quantification

#### 2.8.1 Protein quantification

The total protein content of EVs was quantified using the MicroBCA protein assay kit (Thermo Fisher Scientific, Waltham, United States of America) according to the manufacturer’s instructions.

#### 2.8.2 Nanoparticle tracking analysis

The size and concentration of isolated EVs were measured using a Nanoparticle Tracking Analysis instrument (NanoSight LM10; Malvern Instruments Ltd., Malvern, UK). Data were analyzed using the NTA software (version 2.3, build 0017). To perform the measurements, samples were diluted with PBS and readings were taken in triplicate for 60 s at 10 frames per second at room temperature (25°C). The results were annotated as the number of particles/ml.

### 2.9 Real-time PCR

Reverse transcription was performed with 0.3 μg of total RNA and random primers, with ImProm-II Reverse Transcriptase Kit (Promega, Madison, USA). qPCR was carried out using the GoTaq^®^ Real-Time PCR Systems (Promega) on a StepOne Plus thermal cycler (Applied Biosystems, Waltham, United States of America), according to the recommended protocol. Data were normalized according to Vandesompele et al. (2002), using GAPDH and tubulin as reference genes.

### 2.10 Western blotting

A total of 5 × 10^6^ METAs (for GP82 detection) and 5 × 10^6^ TCTs (for trans-sialidase detection) were centrifuged at 1,000×*g* for 10 min at 4°C and washed with cold PBS buffer, and the pellet was frozen at −20°C. Immediately before being used, the samples were sonicated in a water bath for 15 min and then denatured for 5 min at 95°C. Protein samples were resolved by SDS-PAGE (12% polyacrylamide gel). The total parasite protein extract was applied. The run was performed in a vertical electrophoresis tank in SDS-PAGE buffer. The voltage was fixed at 120 V for 10 min for the stacking gel and 80 V for the separation gel for 3 h. After electrophoresis, the proteins were transferred to a polyvinylidene difluoride (PVDF) membrane in the Trans-Blot SD semi-dry transfer system (Bio-Rad, USA) at 10 V for 40 min. The membrane was incubated overnight with shaking at 4°C with a blocking solution. For the detection of GP82, the membrane was incubated with the primary anti-GP82 antibody [dilution 1:200 in PBST (1% v/v Tween 20)] for 2 h and then with the secondary anti-mouse antibody conjugated to peroxidase at a 1:500 dilution (Santa Cruz Biotechnology, USA) for 90 min. For the detection of trans-sialidases, the membrane was incubated with Mab 39 that recognizes trans-sialidases in bands ranging between 120 and 220 kDa (Schenkman et al., 1992) [dilution 1:200 in PBST (1% v/v Tween 20)] overnight and then with the secondary anti-mouse antibody conjugated to peroxidase at a 1:500 dilution (Santa Cruz Biotechnology, USA) for 90 min. Finally, it was developed on an Amersham™ 600 (GE Healthcare, USA) photo-documenter by chemiluminescence using the Amersham™ ECL™ Prime Western Blotting Systems kit (GE Healthcare, USA). The relative quantification of protein bands in Western blot films was analyzed using ImageJ software.

### 2.11 Dynamics/profile of TCT release by infected cells

Vero cells (5 × 10^4^) were plated for 24–36 h until reaching semiconfluence. The cells were infected with TCTs at different MOIs (indicated in the figure captions) for 24 h and then washed. The supernatant was observed using a Neubauer chamber in order to count the TCTs and extracellular amastigotes released every 24 h, and then the culture medium was changed (replaced with fresh 5% FBS) to continue with the infection until most of the cell culture was destroyed.

### 2.12 Data presentation and statistical analysis

All data are presented as the mean ± standard deviation of at least two independent experiments performed in duplicate or triplicate, unless otherwise reported. The mean values of two groups were analyzed using one-way or two-way analysis of variance (ANOVA), and Tukey’s test was used for multiple comparisons. *p*-values <0.05 were considered statistically significant. Data were analyzed using GraphPad Prism software (version 6.0).

## 3 Results

### 3.1 Exposure to normal human serum selects for a complement-resistant epimastigote population, a phenotype that is transferred to metacyclic forms

**Figure 1:**
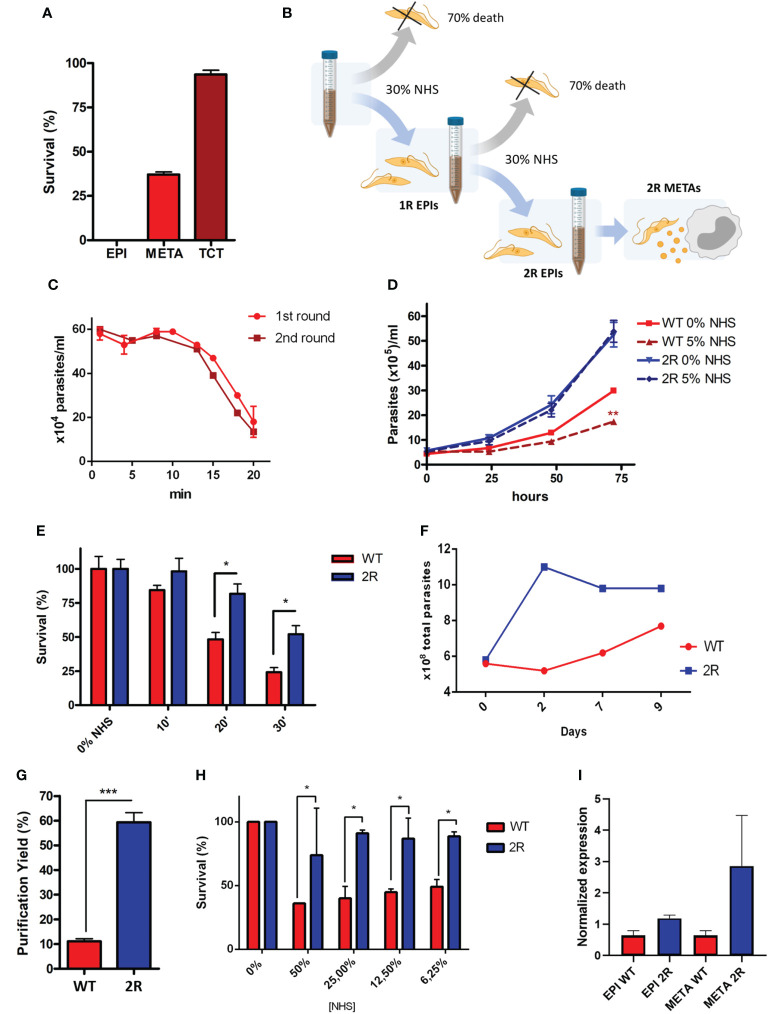
**(A)** Resistance to complement-mediated lysis of different stages of *Trypanosoma cruzi* to 50% normal human serum (NHS) exposition for 30 min. **(B)** Schematic representation of the selection protocol of epimastigote populations (1R and 2R) resistant to complement lysis. The selection protocol is based on a short exposition to high NHS concentrations (30% NHS). **(C)** Parasite survival under 30% NHS exposure during the first and second rounds of selection. **(D)** EPI growth curve in the LIT medium and with the addition of 5% NHS. **(E)** EPI complement-mediated lysis kinetics at 6.25% NHS. **(F)** Viability of parasites (parasites with normal morphology and movement) after metacyclogenesis induction by nutritional starvation (approximately 2.5% FBS final concentration). **(G)** Yield of metacyclic trypomastigote (META) purification by ion exchange chromatography. **(H)** Comparison of complement-mediated lysis resistance at different NHS concentrations for 30 min between META wild type (WT) and 2R. **(I)** Expression of CRIT by epimastigotes and METAs of WT and 2R evaluated by quantitative real-time PCR. * P<0.05. *** P<0.001.

During the life cycle of *T. cruzi*, the parasite undergoes morphological and surface composition changes, resulting in higher degrees of resistance to complement-mediated lysis and infectivity in METAs and TCTs. To compare the resistance to complement system lysis between different stages from the same strain (G), we exposed EPI, META, and TCT forms to physiological (host) NHS concentrations (50%) for 30 min. As expected, we have seen that the infective forms have a higher resistance to complement-mediated lysis (37% METAs and 93.6% TCTs) compared with EPIs, which were fully lysed in this condition (**Figure 1A**).

We hypothesized whether it would be possible to select a population of EPIs more resistant to NHS and characterize its phenotype after metacyclogenesis. For this, we performed a survivor selection protocol in two rounds of short exposure to NHS (illustrated in **Figure 1B**; see *Methods*). We exposed log-phase EPIs to NHS and followed the lysis of the population until 30% survival, which occurred in 20 min (**Figure 1C**). The remaining population was recovered after the completion of a growth curve and was again exposed to lysis under the same conditions (30% NHS to 30% survival). The survivor population was recovered and named 2R. Parasites from the 2R selected population, once established, demonstrated an accelerated growth curve when compared with the WT population and, in the presence of 5% NHS, decreased the growth of WT parasites, but not 2R (**Figure 1D**). Furthermore, 2R EPIs showed a higher survival to complement lysis when compared with WT (**Figure 1E**).

After the resistant population was selected, we were interested in analyzing the virulence of the selected population in the metacyclic stage, which is responsible for the first contact with the mammalian host. Therefore, the populations of WT and 2R were submitted to the process of metacyclogenesis, which is experimentally induced by nutritional stress/starvation. During differentiation, 2R parasites were more viable compared with WT (**Figure 1F**), allowing a greater purification of METAs in the 2R population by ion exchange chromatography (**Figure 1G**). Moreover, the population of 2R METAs remained more resistant to complement-mediated lysis compared with WT, in all tested NHS concentrations (**Figure 1H**), showing that the resistant phenotype could be transferred to the metacyclic stage. The higher expression of CRIT (one of the molecules that participates in parasite-mediated complement lysis evasion) by 2R METAs could partially explain its resistance phenotype (**Figure 1I**).

### 3.2 METAs from the selected population are more invasive of the host cells

**Figure 2:**
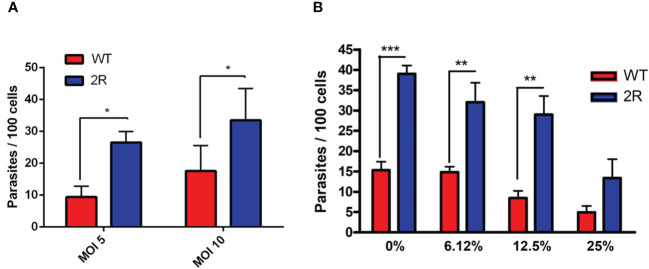
**(A)** Invasion assays of WT and 2R METAs in Vero cells proceeded at a multiplicity of infection (MOI) of 5 and 10 for 3 h. **(B)** Invasion assays of WT and 2R METAs in Vero cells executed in the presence of different concentrations of NHS for 3 h (MOI 10). * P<0.05. ** P<0.01. *** P<0.001.

Our group demonstrated that the human complement system has a limited capacity to lyse metacyclic parasites, allowing the infection of host cells and the intracellular life of the parasite (Cestari and Ramirez, 2010). Considering this evidence, we decided to analyze the invasiveness of the selected parasite population to mammalian cells. Surprisingly, METAs from the 2R population were at least twice more invasive than WT at an MOI of 10 and 5 (**Figure 2A**). Moreover, NHS could not control the infection since the 2R-resistant population invaded at least two times more than WT META forms in all the different NHS concentrations (**Figure 2B**). As GP82 is the main surface molecule related to *T. cruzi* META invasion, we performed a Western blot in which META total protein extracts were incubated with anti-GP82 antibodies. In this experiment, a more intense band of 82 kDa was observed in 2R META compared with WT parasites (**Supplementary Figure 1**), which could suggest that GP82 is expressed more in the resistant population.

### 3.3 VEs! from the META 2R population were capable of transferring a most invasive phenotype to WT parasites

**Figure 3:**
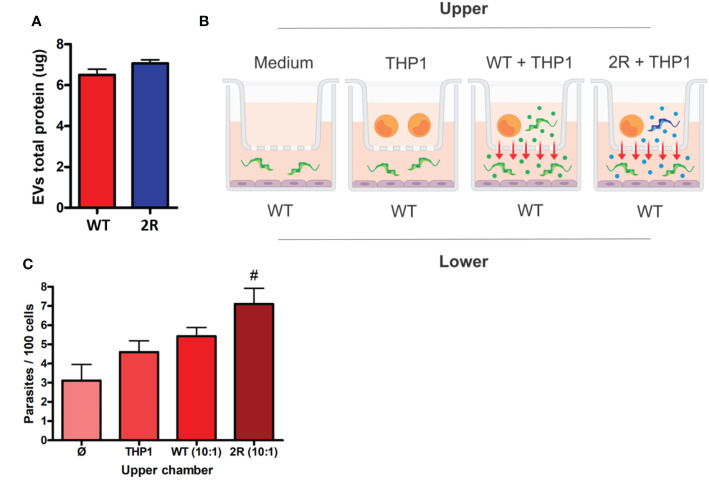
**(A)** Protein dosage of EVs secreted by METAs in the interaction with THP-1 cells (5 parasites for each cell). **(B)** Schematic representation of the Transwell assay. Two chambers are separated by a membrane with 0.45 µm porosity. In the lower chamber, an invasion test is performed that receives EVs from the upper layer. **(C)** Invasion rates of WT METAs to Vero cells during the Transwell assay. The *x*-axis labels indicate what was placed in the upper chamber of the Transwell plate. # indicates *p <*0.01 in comparison to control (only medium in the upper chamber—*Ø*).

As *T. cruzi* EVs are being described as the mechanisms of virulence and evasion of immune effector mechanisms (Rossi et al., 2021), we analyzed EVs derived from the contact of host cells with WT and 2R parasites that could contain membrane from both organisms. It was found that in contact with THP-1, METAs from both populations produced similar amounts of EVs (**Figure 3A**). We were interested in analyzing whether EVs of the selected population were capable of transferring the phenotype of higher invasiveness to WT parasites. To determine this, we performed co-culture experiments incubating METAs and THP-1 cells in the upper chamber of a Transwell plate. The lower chamber was used to carry out an invasion assay between semiconfluent Vero cells and WT META (**Figure 3B**; see *Methods*). Interestingly, we found that EVs released during the interaction between 2R METAs and THP-1 cells were able to promote greater invasion of WT parasites than EVs from WT METAs (**Figure 3C**).

### 3.4 The virulence/infectivity phenotype of the selected population remains in TCT forms

**Figure 4:**
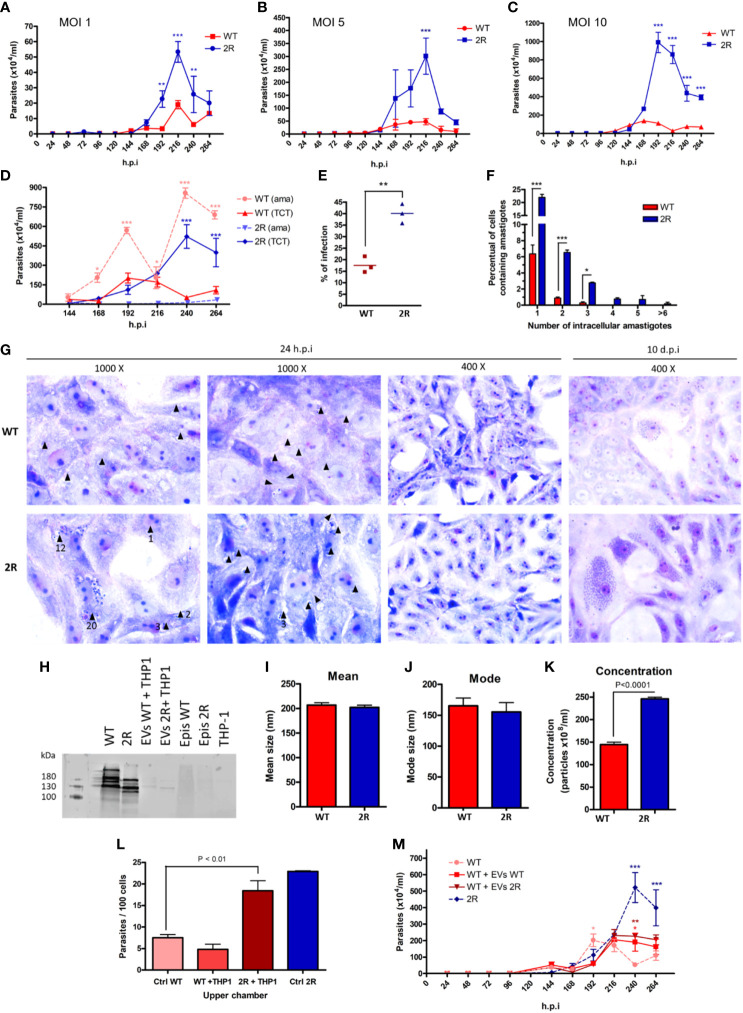
Parasite count in the supernatant after infection of Vero cells with TCTs at MOIs of 1 **(A)**, 5 **(B)**, and 10 **(C)**. **(D)** Comparison between the release of extracellular amastigotes (ama) and TCTs in the supernatant of cells infected at an MOI of 10. **(E)** Percentage of infected Vero cells after 24 h of infection with TCTs and another 24 h of incubation (MOI of 5). **(F)** Intracellular amastigotes counting 24 h.p.i. (MOI of 5). **(G)** Micrographs of Vero cells infected by TCT WT or 2R 24 h.p.i. (left panels) or 10 d.p.i. (last right panel) stained by Giemsa. Black arrows indicate the presence of amastigotes, and the numbers below the arrows indicate the amount of amastigotes (if present). After 10 days, the infection of Vero cells by WT TCTs (upper panel) has few cells with a large amount of amastigotes when compared with 2R TCTs (lower panel). **(H)** Analysis of the expression of trans-sialidases in TCTs of WT and 2R using antibody Mab 39 (bands ranging between 120 and 220 kDa). Samples analyzed: TCT WT (lane 1), TCT 2R (lane 2), 11 μg EV TCT WT with THP-1 (lane 3), 11 μg EV TCT 2R with THP-1 (lane 4), epimastigotes WT (lane 5), epimastigotes 2R (lane 6), and THP-1 (lane 7). **(I, J)** Mean and mode size of EVs derived from the interaction of TCTs with THP-1 in a 5 to 1 ratio (parasites to cells). **(K)** Concentration of EVs released from the interaction of TCTs with THP-1 in a 5 to 1 ratio (parasites to cells). **(L)** Invasion rates of WT TCTs to Vero cells during Transwell assay. The *x*-axis labels indicate what was placed in the upper chamber of the Transwell insert. “Ctrl WT” refers to the infection of WT parasites in only media. **(M)** Release of TCTs in the supernatant of infected cells at an MOI of 10 in the presence of 30 µg of EVs during the first 24 h of infection. * P<0.05. ** P<0.01. *** P<0.001.

Interested in evaluating the phenotype among the different evolutionary forms of the parasite, we compared infectivity characteristics of trypomastigotes derived from cell culture (TCTs) of the two populations. Vero cells were infected by TCTs in different MOIs (1, 5, and 10), and the release of parasites was monitored every 24 h by counting the supernatant of the infection and then changing the cell medium. In all MOIs, a greater release of TCTs from the selected population was observed in comparison with WT, which occurred from 192 to 240 h.p.i. (**Figures 4A-C**). A striking observation was the high release of amastigotes in WT infections, which was up to 16 times greater than the number of TCTs in the infection supernatant (**Figure 4D**). As also seen in METAs (**Figure 2A**), the selected parasites in TCT forms proved to be more infectious than the WT population at 48 h.p.i. (**Figure 4E**). Moreover, it was also seen that 2R had a higher rate of replication of amastigotes within infected cells in just 24 h.p.i. (**Figures 4F, G**). After 10 days of infection, the presence of infected cells was much higher in the 2R population (**Figure 4G**, last panel). Interestingly, the parasites of the selected population showed a different pattern of expression of trans-sialidases on their surface, an enzyme related to evasion of the immune system, infectivity to host cells, and pathogenicity of *T. cruzi* (**Figure 4H**).

We analyzed EVs derived from TCTs in contact with THP-1 cells and found that the selected population was able to induce a greater amount of EVs than WT, despite the EVs of both being similar in mean size and mode (**Figures 4I-K**). As seen in METAs, the EVs derived from the selected population were able to increase the infection of WT parasites (**Figure 4L**). The treatment of Vero cells with EVs during the time of infection allows a prolonged release of TCTs from the WT strain (**Figure 4M**).

## 4 Discussion

### 4.1 Differences in resistance to complement-mediated lysis between stages are related to several evasion mechanisms

During its life cycle, *T. cruzi* faces multiple regulated morphological and physiological modifications to survive within the triatomine vector and mammalian host cells. While epimastigotes are highly susceptible to complement-mediated lysis, metacyclic trypomastigotes display a certain resistance to complement lysis, enough to establish the infection due to the rapid invasion capacity of the parasite (Cestari et al., 2008). Bloodstream trypomastigotes are even more resistant to elimination by the complement system, and the adaptive immune response gains greater importance through the course of infection, when cells and cytokines define the control of parasitemia and the development of pathology (Acevedo et al., 2018; Cristovão-Silva et al., 2021). In the bloodstream, *T. cruzi* resists lysis mediated by the complement system with the support of its wide repertoire of surface proteins (Lidani et al., 2017) by capturing host plasma molecules (Pangburn, 2000) or shedding EVs (Cestari and Ramirez, 2010).

### 4.2 *Trypanosoma cruzi* intrapopulation diversity allows the selection of parasites *in vitro* or *in vivo* after exposure to some stressor agents

Based on our hypothesis, within a population of *T. cruzi*, subpopulations with different “skills” or virulence may have a greater chance of surviving when exposed to a different environment. With our approach, it was possible to select a subpopulation of parasites that seemed to have a more prominent resistance characteristic to complement-mediated lysis (**Supplementary Figure 2**). This diversity could be explained considering that *T. cruzi* has a complex genomic architecture that allowed, during its evolution, the accumulation of discrete mutations (Tibayrenc and Ayala, 2002; De Araujo et al., 2020), leading to the heterogeneity of populations, even among an individual strain (Manoel-Caetano and Silva, 2007; Llewellyn et al., 2015; Seco-Hidalgo et al., 2015). In fact, this high variability has already been shown in experimental (*in vitro* and *in vivo*) and clinical studies of Chagas disease. Samples collected at two points in a patient’s life were different in terms of the electrophoretic profile due to the digestion of kinetoplast DNA and the isoenzyme pattern (de Lana et al., 1996), suggesting that, once infected, the host’s immune system is capable of selecting a population of parasites. Experimentally, Murta and Romanha (1998) were able to select a population of *T. cruzi* resistant to benznidazole after 25 successive passages in mice treated with a single high dose of the drug (Murta and Romanha, 1998). Other studies have shown that it is possible to select a population more resistant to drugs with a protocol of long exposure of parasites *in vitro*, and importantly, this resistance was maintained even after differentiating the parasites to other evolutionary forms (Nirdé et al., 1995; Mejia et al., 2012). These reports, together with our data, reinforce, first, that there is a high intrapopulation variability in *T. cruzi* and that exposure to a stressor is able to eliminate sensitive parasites and preserve resistant ones, which multiply and end up predominating in the population.

### 4.3 The phenotype of selected epimastigotes is transferred to infective stages

Studies have drawn attention to the plasticity that microorganisms have to ensure infection in different hosts (Cameron et al., 2013; Leggett et al., 2013; Searle et al., 2015; Zimmermann et al., 2017; Dumoulin and Burleigh, 2018; Schneider et al., 2018; Quintana et al., 2021; Schuster et al., 2021). A life cycle as complex as that of *T. cruzi*, involving very different environments and hosts, requires great morphological and biochemical plasticity of evolutionary forms (de Godoy et al., 2012; Li et al., 2016; Queiroz et al., 2016; Belew et al., 2017; Gonçalves et al., 2018). Although the transition from epimastigotes to metacyclic trypomastigotes is better understood (metacyclogenesis) (Parodi-Talice et al., 2007; Ferreira et al., 2008; Gonçalves et al., 2018), the intracellular transformation of amastigotes into blood trypomastigotes is still poorly understood. The work by Belew et al. (2017) has provided a comparative transcriptome analysis of two cloned *T. cruzi* strains (CL Brener and CL-14) that display contrasting virulence phenotypes. Gene expression analysis during the intracellular cycle has indicated that the avirulent phenotype of CL-14 may be due, at least in part, to a reduced or delayed expression of genes encoding surface proteins that are associated with the transition from amastigotes to trypomastigotes. Our data indicate that adaptations suffered by the parasite at a replicative stage are maintained and may persist during its life cycle promoting an evolutionary impact on the infection.

### 4.4 Extracellular vesicles can transfer the ability to invade host cells

EVs are key players in intercellular communication without direct cellular contact, due to their ability to alter the phenotype or modulate the gene expression of target cells (Raposo and Stahl, 2019). Several studies have suggested that EVs play an important role in parasite–host cell dynamics and in the physiopathology of Chagas disease (reviewed by de Pablos Torró et al., 2018 and Rossi et al., 2019). Here, we have analyzed EVs derived from the contact of *T. cruzi* with THP-1 cells, resulting from an intense dynamic of EV release during the host–parasite interaction. We found that EVs released by 2R METAs in contact with THP-1 cells were able to promote greater invasion of WT parasites than EVs from WT METAs. Furthermore, the Transwell assays reinforce the idea of the dynamic releasing of EVs during the contact of parasites and host cells that contribute to a higher invasion phenotype. This result suggests that EVs could transfer a phenotype of greater invasiveness to a less infective parasite. It is known that *T. cruzi* EVs are capable of inhibiting complement attack (Cestari et al., 2012) and increasing the infection of host cells by parasites (Neves et al., 2014; Ramirez et al., 2017; Wyllie and Ramirez, 2017; Ribeiro et al., 2018). Furthermore, some *in-vivo* studies reinforce that EVs play a pro-parasitic role, increasing parasitemia and the severity of the pathology when animals are infected in the presence of EVs (Torrecilhas et al., 2009; Cestari et al., 2012; Ramirez et al., 2017). Here, our group confirms the participation of EVs in the complex scenario of *T. cruzi* infection, in which EVs from virulent parasites were able to transfer a more aggressive phenotype to the WT population.

## Data availability statement

The raw data supporting the conclusions of this article will be made available by the authors, without undue reservation.

## Author contributions

IR designed the study, conducted the experiments, and wrote and edited the manuscript. MN conducted the experiments. BS conducted the experiments and edited the manuscript. HR, SW, and AR conducted the experiments. JI edited the manuscript. MR designed the study, conducted the experiments, and wrote and edited the manuscript. All authors contributed to the article and approved the submitted version.
