# Supplementary figures and images for: An induced population of Trypanosoma cruzi epimastigotes more resistant to complement lysis promotes a phenotype with greater differentiation, invasiveness, and release of extracellular vesicles
Source: Front Cell Infect Microbiol. 2022 Dec 14;12:1046681. doi: 10.3389/fcimb.2022.1046681 (PMC9795005; doi:10.3389/fcimb.2022.1046681)

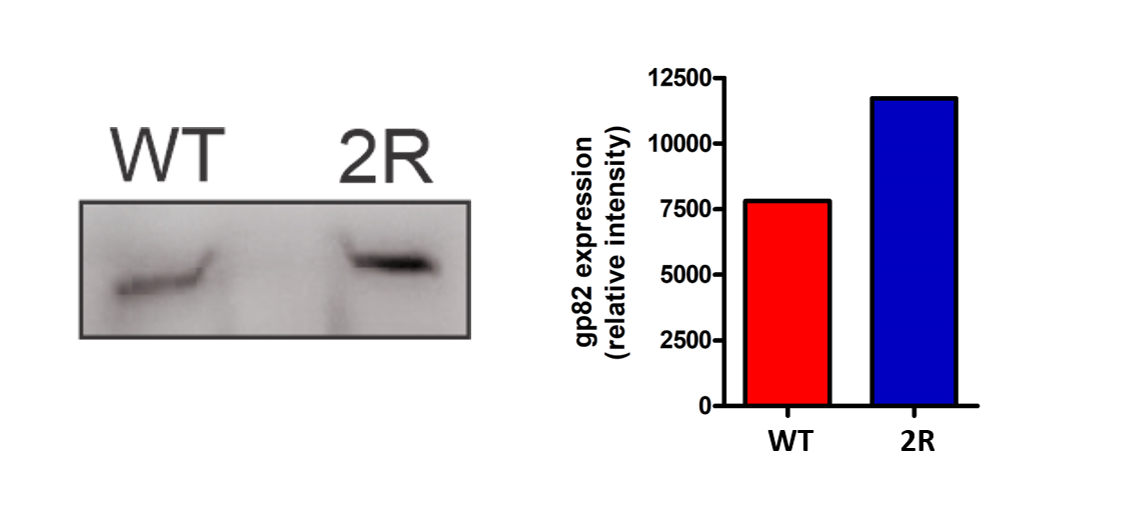

Supplement: Supplementary file 1 [file Image_1.tif]

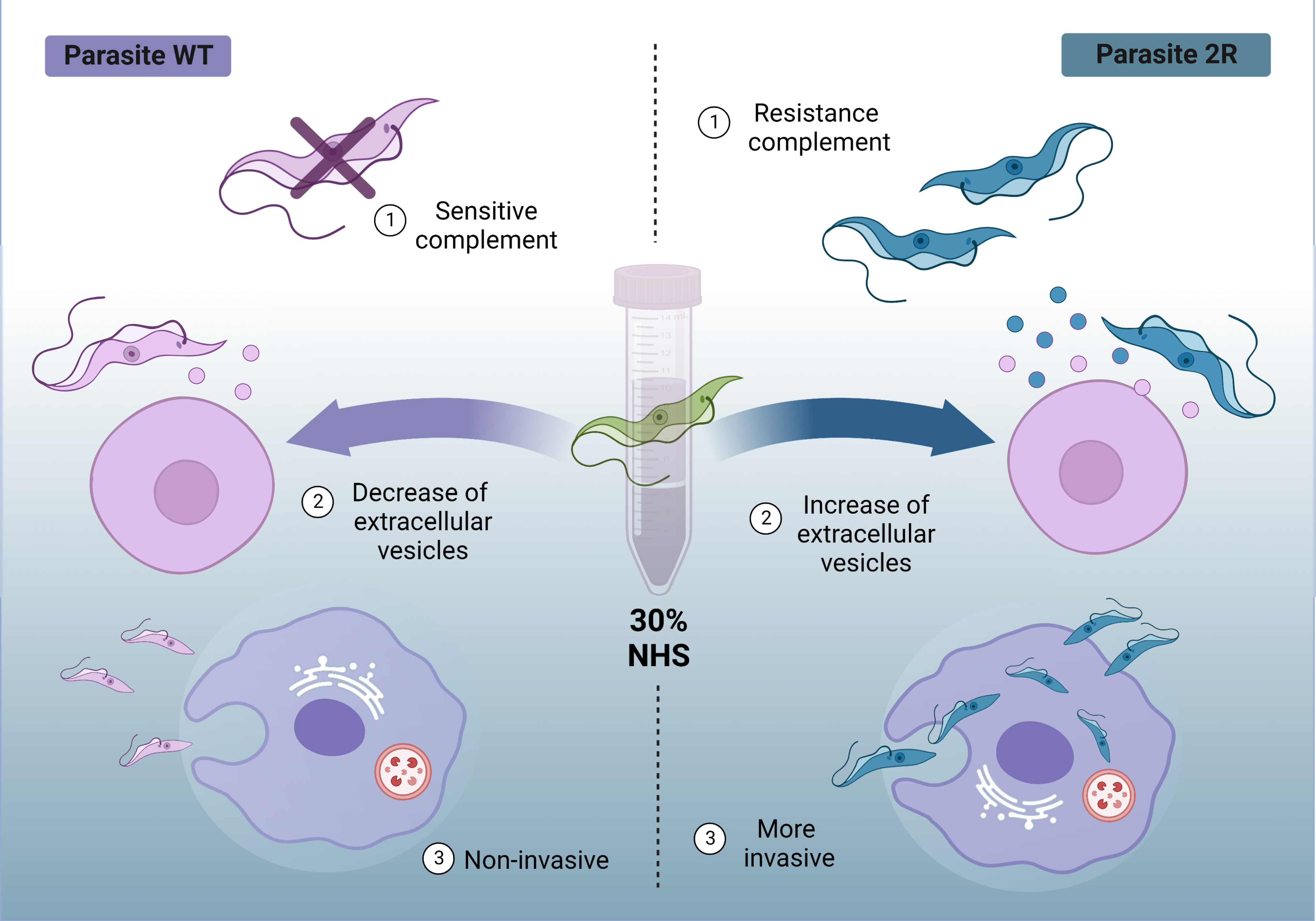

Supplement: Supplementary file 2 [file Image_2.tif]
